# The Comparison of Aspirin and Direct Oral Anticoagulant as Thromboprophylaxis Following Total Knee Replacement: A Retrospective Study

**DOI:** 10.1055/s-0044-1785510

**Published:** 2024-12-21

**Authors:** Endrotomo Sumargono, Maria Anastasia, Leonard Christianto Singjie, Ifran Saleh, Ira Juliet Anestessia, Erica Kholinne

**Affiliations:** 1 Departamento de Cirurgia Ortopédica, St. Carolus Hospital, Jakarta, Indonésia; 2 Faculdade de Medicina, Universitas Katolik Indonesia Atma Jaya, Indonésia; 3 Departamento de Cirurgia Ortopédica, Faculdade de Medicina, Universitas Indonesia, Jakarta, Indonésia; 4 Faculdade de Medicina, Universitas Trisakti, Jakarta, Indonésia

**Keywords:** anticoagulants, arthroplasty, replacement, knee, venous thromboembolism

## Abstract

**Objective** Venous thromboembolism (VTE) is still a major challenge after major orthopaedic surgery, including total knee replacement (TKR). The aim of this study was to estimate the risk of VTE with aspirin-only pharmacologic prophylaxis following primary TKR surgery versus direct oral anticoagulant (DOAC).

**Methods** The study included 476 patients who underwent primary TKR from 2016 to 2020. All patients received thromboprophylaxis with DOAC (DOAC group) ( *n* = 267) or aspirin (aspirin group) ( *n* = 209). Clinical outcomes were evaluated and compared between those who received DOAC and aspirin. The primary outcome was the incidence of VTE. The secondary outcome was wound complications.

**Result** Aspirin and DOAC were comparable in preventing VTE in patients who underwent primary TKR. The incidence of deep vein thrombosis was similar in the aspirin (10%) and factor Xa inhibitor groups (10.1%), ( *p* = 0.98) with zero case of pulmonary emboli in both groups. There was no significant difference between the aspirin (1.4%) and DOAC groups (1.5%) regarding wound complication ( *p* = 0.95).

**Conclusion** Postoperative thromboprophylaxis with aspirin only was not associated with a higher risk of postoperative VTE compared with DOAC following TKR. Considering the wide availability and cost-effectiveness, aspirin may serve as a promising alternative to DOAC for VTE prophylaxis.

## Introduction

Total knee replacement (TKR) has been one of the most successful orthopaedic interventions which have an impact on improving the quality of life. Projection studies suggest that the demand for primary TKR will steadily increase in the years to come. 1 However, the TKR procedure also has some postoperative complications. One of the most common yet serious complications is venous thromboembolic disease (VTE). Venous thromboembolism is a known complication after major orthopaedic surgery, including knee or hip replacement. 2 3

Several studies have examined the efficacy of several antithrombotic drugs over their years as well as their side effects profiles, including prolonged wound leakage, bleeding, and infection. 4 5 The following agents that are usually used for thromboprophylaxis are antiplatelet (aspirin) or direct oral anticoagulant (DOAC), such as factor Xa inhibitor (rivaroxaban or edoxaban). However, it is currently recognized that aspirin is preferable over other anticoagulants because of its low risk of inducing major bleeding. 6 7 Aspirin may serve as an alternative thromboprophylaxis agent following primary knee or hip replacement, particularly in patients at high risk of bleeding. Other advantages of aspirin over DOACs include its ease of administration, lack of monitoring needs, and low cost. 8

Indonesia is one of the lower-middle income countries according to the World Bank. 9 Therefore, there is a need to find the most effective and safe thromboprophylaxis with the most reasonable cost. The current study aims to assess the clinical effectiveness and safety of aspirin compared with DOAC for VTE prophylaxis after TKR in a single institution. We hypothesized that there was no significant difference between aspirin and DOAC in preventing VTE after TKR procedure.

## Methods

### Data Source and Study Cohort

**Fig. 1:**
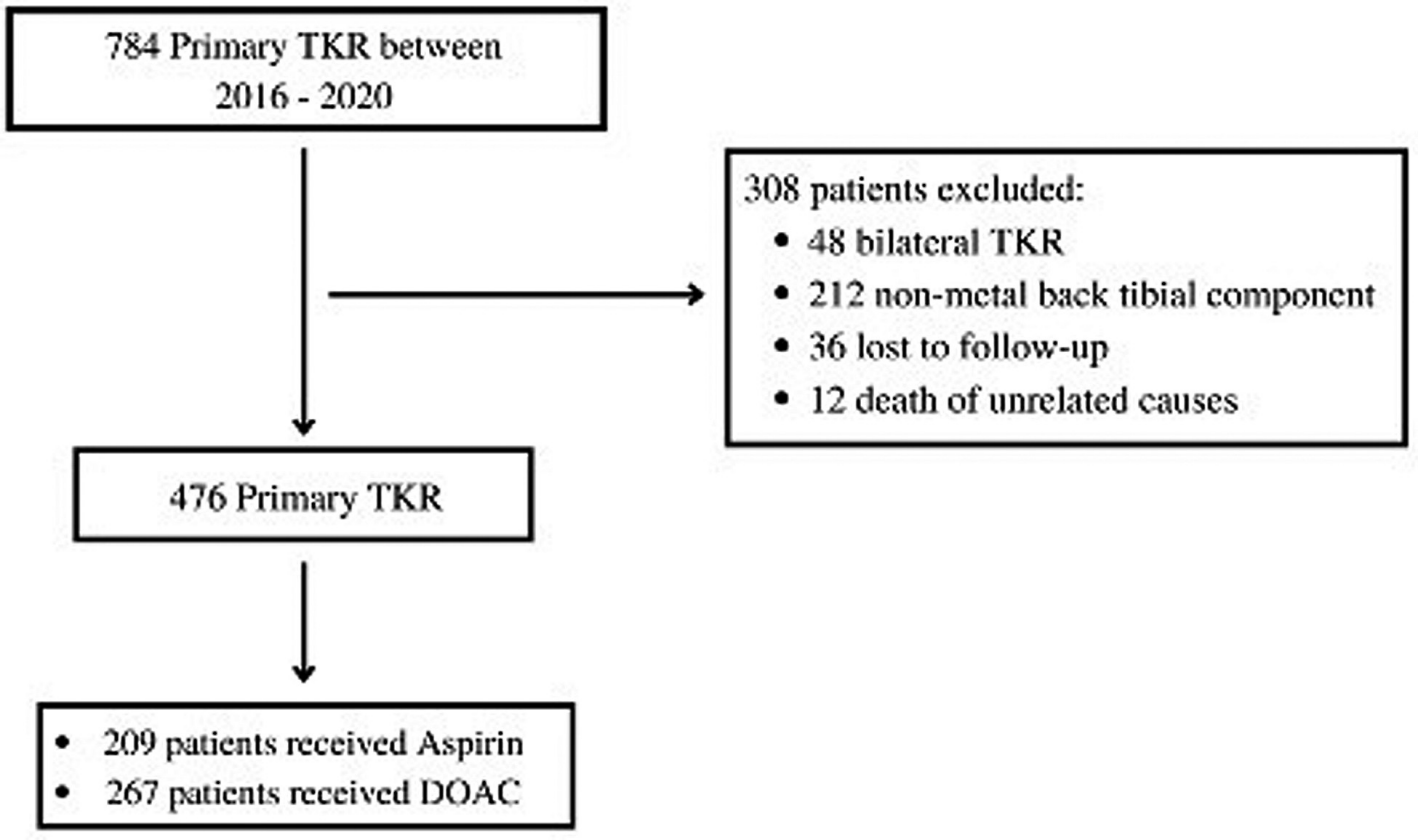
Flow chart of study sample selection.

This Institutional Review Board (IRB) of our institution approved the present study under the number (Number 940/RSSC-5B/AL/IX/DIRUT/2022). Retrospective data of 476 primary TKRs was extracted from the 5 years (2016–2020) of annual reports of TKR registry data in our institution. The inclusion criteria were as follows: (1) patients who underwent primary TKR surgery; and (2) patients who received aspirin or DOAC for VTE prophylaxis. The exclusion criteria were as follows: (1) Prior hemorrhagic disease; (2)history of hypercoagulation state; (3) history of malignancy; (4) history of venous insufficiency; (5) bilateral TKR; (6) non-metal back tibial component. The sample selection of the study is shown in Fig. 1 .

The primary outcome was postoperative symptomatic venous thromboembolism (VTE) occurring during the index hospitalization, or responsible for readmission within 30 days, or an outpatient visit within 90 days after discharge, because an increased thromboembolic risk persists up to 3 months postoperatively. The secondary outcome was wound complication. All patients underwent the same surgical technique (medial parapatellar approach and application of a tourniquet 150 mmHg above diastolic blood pressure) and perioperative protocol (perioperative pain medication, 2 g of intravenous cefazoline as prophylactic antibiotics. The mechanical prophylaxis was periodic ankle pumping, started 6 hours postoperatively, along with the application of an intermittent pneumatic compression device (IPCD). All patients received chemical thromboprophylaxis, which were aspirin 160 mg aspirin group) or factor Xa inhibitors (DOAC group), which was either rivaroxaban 10 mg (Xarelto; Bayer Pharmaceutical, Leverkusen, Germany) or edoxaban 30 mg (Lixiana; Kalbe Farma, Jakarta, Indonesia) 12 hours following surgery and continued for 14 days. All of the implants used in this study were PFC Sigma or Attune (DePuy Synthes, Raynham, MA, USA). Investigations for deep vein thrombosis (DVT) were initiated on symptomatic patients in the routine follow-up examination. The diagnosis of VTE was made by confirming thrombosis of the deep vein involving the popliteal vein or more proximal leg veins (including the femoral, common femoral, and iliac veins) with a sonogram by a radiologist. Wound complication was defined as patients requiring irrigation and debridement, with or without component exchange. All data were collected from medical records by two medical doctors posted in the orthopedic service who were not involved in the surgery.

### Statistical Analysis

All of the experimental data were statistically analyzed using SPSS Statistics for Windows version 26.0 software (IBM Corp, Armonk, NY, USA). Bivariate analysis was used to test the association between individual thromboprophylaxis and the major clinical outcomes of postoperative VTE using the Chi-squared test. Statistical significance was set at *p* < 0.05.

## Result

The demographic data in Table 1 showed that there were no significant demographic differences between the two groups. The gender majority in both groups was female (80% versus 81%). More than ⅔ of the patients in both groups had comorbidities. Both groups showed similar comorbidities profiles, with hypertension being the most common comorbidity (45,4% and 51,3% in the aspirin and the DOAC groups, respectively). The length of stay in the aspirin and DOAC groups was equivalent (4.4 versus 4.6 days).

**Table 1:** Patients' d data

Characteristic	Aspirin group N = 209 (45%)	DOAC group N = 267 (55%)	*P* -value
Mean age (years)	68.3	67.9	0.32
Gender: male/female (%)	43/166 (20/80)	53/214 (19/81)	0.06
Comorbidities			0.94
Hypertension (%)	95 (45,4)	137 (51,3)	
Diabetes mellitus type 2 (%)	32 (15,3)	39 (14,6)	
Cardiovascular disease (%)	29 (13,8)	28 (10,4)	
Kidney disease (%)	7 (3,3)	2 (0,7)	
Cerebrovascular disease (%)	3 (1,4)	3 (1,1)	
Liver disease (%)	3 (1,4)	3 (1,1)	
Mean length of stay (SD)	4.4 (2.7)	4.6 (2.1)	0.09

Abbreviation: DOAC, direct oral anticoagulant.

**Fig. 2:**
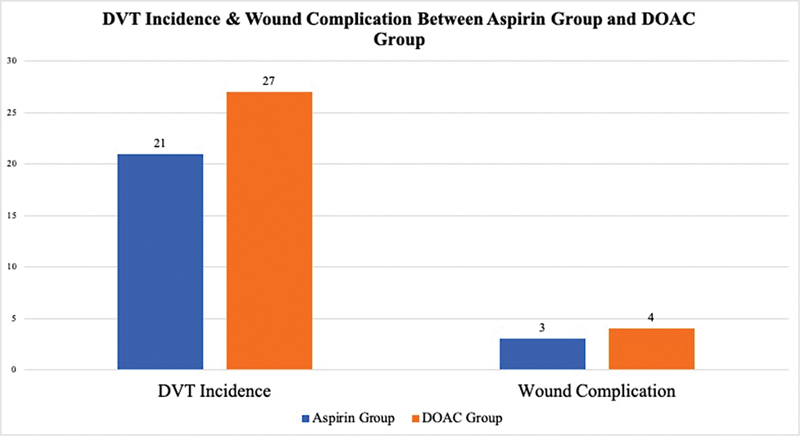
Incidence of deep vein thrombosis (DVT) & wound complication in the Aspirin and direct oral anticoagulant (DOAC) groups.

There were 21 DVT cases (10%) in the aspirin group compared with 27 cases (10.1%) in those in DOAC group ( *p* = 0.98). For wound complications, there were 3 cases in the aspirin group (1.4%) and 4 cases in the DOAC group (1.5%); ( *p* = 0.95). Only 2 cases required component exchanges because of prosthetic joint infection, 1 from each group. The rest were treated with irrigation and debridement. Fig. 2 showed that there was a similar number of DVT incidence and wound complications after TKR between the aspirin and DOAC groups.

## Discussion

From our study, we found that aspirin and the DOAC (rivaroxaban or edoxaban) were comparable in preventing VTE in patients who underwent TKR. The incidence of DVT was similar in the aspirin and factor Xa inhibitor groups (10% versus 10.1%, respectively), with no occurrence of pulmonary emboli (PE) in either group. There was no association between the type of VTE prophylaxis agent with the risk of wound complication in post-TKR patients because the incidence in the aspirin and factor Xa inhibitor groups (1.4% versus 1.5%, respectively) was also similar.

The exact incidence of DVT and pulmonary emboli remains controversial. 10 Without any prophylaxis protocol, incidence rates greater than 50% following joint replacement have been reported. 11 The incidence has dramatically decreased nowadays, with early mobilization, mechanical compression devices, and chemical prophylaxis. 11 There has been substantial debate regarding the preferred chemical prophylactic agent among aspirin, enoxaparin, warfarin, and DOAC when weighing VTE prophylaxis efficacy versus bleeding risk. 12 Direct oral anticoagulants have a better VTE prophylaxis compared with other anticoagulants, with a lower rate of complications, such as the need for blood transfusion and prolonged wound drainage. 13 Aspirin is commonly used for primary or secondary prevention of cardiovascular diseases, as it inhibits the aggregation of platelets by the irreversible acetylation of cyclooxygenase, thus interfering with the ability of platelet to produce prostaglandin. Aspirin also provided a comparable VTE prophylaxis to that of DOACs. 12 14 Recently, some studies recommended the use of aspirin as another option for VTE prophylaxis, in addition to DOAC or low-molecular-weight heparin (LMWH) for elective joint replacement surgery, including TKR, combined with mechanical prophylaxis until hospital discharge. 15

The current study showed that the VTE incidence was not influenced by the type of thromboprophylaxis used is supported by recent literatures. Several studies also concluded that the use of aspirin following TKR was associated with low rates of thromboembolic events, similar to the rates of those treated with DOAC. 14 15 Aspirin has been found to have a low VTE rate, with no difference when compared with enoxaparin following TKR procedure. 16 Venker et al. 17 showed that DOAC was superior to enoxaparin in preventing DVT. Other randomized clinical trials by Le et al. 18 and Xu et al. 19 reported that there was no difference between DOACs (rivaroxaban) and aspirin when used as VTE prophylaxis following TKR, which supported the finding of the current study. Administration of VTE prophylaxis also comes with several drawbacks, such as postoperative bleeding and prolonged wound drainage. 19 Postoperative bleeding is one of the major concerns of VTE prophylaxis. The more potent the pharmacologic agent, the higher the risk it poses. 20 Aspirin has been found to have a lower bleeding risk compared with other VTE prophylaxis agents, as reported by Wilson et al. 21 Similarly, DOAC also has a low bleeding risk compared with enoxaparin and warfarin, especially if given in a low dose. 13 Another disadvantage of VTE prophylaxis is postoperative wound complication. A study conducted by Lassen et al. 22 reported that administration of rivaroxaban increased wound complications following TKR, although not as severe as those found in patients using low molecular weight Heparin (LWMH), as reported by Kulsrestha et al. 23 Singh et al. 24 have demonstrated that the use of aspirin lowers the rate of prolonged wound drainage in post-TKR patients, hence, lower the risk of developing any possible wound complication, including superficial and deep wound infection. Comparably, another study by Garfinkel et al. 25 reported that patients who received DOAC, such as apixaban, for VTE prophylaxis following joint replacement had a significantly higher rate of wound complications and bleeding. Interestingly, the current study found that there was no statistical difference between aspirin and DOAC in terms of wound complications.

Aspirin is a generic, inexpensive, and widely-available anti-platelet drug. Hence, the use of aspirin as a VTE prophylaxis can be such a cost-effective choice. 26 As previously stated, Indonesia still comes under the lower-middle income countries, as reported by the World Health Organization. According to the e-catalogue from the Ministry of Health, the cost of aspirin is Rp 116,-/tablet (0.79¢); meanwhile, the price of rivaroxaban is Rp 23.500,-/tablet ($1.39). 27 Therefore, given the comparable effect to DOAC as a thromboprophylaxis agent, aspirin remains the most cost-effective thromboprophylaxis agent, especially when compared with factor Xa inhibitor.

There are a few limitations to this study. First, concerning the nature of a retrospective study, the strength of evidence is still limited. The findings still need to be confirmed with future prospective trials with a higher level of evidence. Second, there are inherent selections and observer biases as patients were from a single hospital. Despite these limitations, our findings are clinically important. The samples in our study are large; therefore, they can be adequate to show aspirin is not inferior compared with rivaroxaban. Future multi-center trials are required to reduce the biases.

## Conclusion

Patients undergoing major orthopedic surgery, such as TKR, are at the highest risk for developing VTE during and after hospitalization. The current study showed that there was no significant difference between aspirin and DOAC in preventing VTE following TKR. When choosing the VTE prophylaxis in patients undergoing TKR surgery, given the safety, wide availability, and cost-effectiveness, aspirin may be considered as a promising alternative to DOAC for the VTE prophylaxis.

## References

[1] Skou S T Roos E M Laursen M B A randomized, controlled trial of total knee replacement N Engl J Med 2015 373 17 1597 1606 26488691 10.1056/NEJMoa1505467

[2] Sloan M Premkumar A Sheth N P Projected volume of primary total joint arthroplasty in the U.S., 2014 to 2030 J Bone Joint Surg Am 2018 100 17 1455 1460 30180053 10.2106/JBJS.17.01617

[3] Healy W L Della Valle C J Iorio R Complications of total knee arthroplasty: standardized list and definitions of the Knee Society Clin Orthop Relat Res 2013 471 01 215 220 22810157 10.1007/s11999-012-2489-y PMC3528930

[4] Lieberman J R Pensak M J Prevention of venous thromboembolic disease after total hip and knee arthroplasty J Bone Joint Surg Am 2013 95 19 1801 1811 24088973 10.2106/JBJS.L.01328

[5] Sasaki H Ishida K Shibanuma N Retrospective comparison of three thromboprophylaxis agents, edoxaban, fondaparinux, and enoxaparin, for preventing venous thromboembolism in total knee arthroplasty Int Orthop 2014 38 03 525 529 24100922 10.1007/s00264-013-2132-x PMC3936088

[6] Lewis C G Inneh I A Schutzer S F Grady-Benson J Evaluation of the first-generation AAOS clinical guidelines on the prophylaxis of venous thromboembolic events in patients undergoing total joint arthroplasty: experience with 3289 patients from a single institution J Bone Joint Surg Am 2014 96 16 1327 1332 25143492 10.2106/JBJS.M.00503

[7] Matharu G S Kunutsor S K Judge A Blom A W Whitehouse M R Clinical Effectiveness and safety of aspirin for venous thromboembolism prophylaxis after total hip and knee replacement: A systematic review and meta-analysis of randomized clinical trials JAMA Intern Med 2020 180 03 376 384 32011647 10.1001/jamainternmed.2019.6108 PMC7042877

[8] Raphael I J Tischler E H Huang R Rothman R H Hozack W J Parvizi J Aspirin: an alternative for pulmonary embolism prophylaxis after arthroplasty? Clin Orthop Relat Res 2014 472 02 482 488 23817755 10.1007/s11999-013-3135-z PMC3890197

[9] Worldbank Indonesia | Data, (n.d.)Available from:https://data.worldbank.org/country/ID. [Accessed October 17, 2022]

[10] The ICM-VTE Hip & Knee Delegates Recommendations from the ICM-VTE: Hip & Knee[published correction appears in J Bone Joint Surg Am. 2022 Aug 3;104(15):e70]J Bone Joint Surg Am 2022 104 01 180 231 35315610 10.2106/JBJS.21.01529

[11] Etzioni D A Lessow C Bordeianou L G Venous Thromboembolism after Inpatient Surgery in Administrative Data vs NSQIP: A Multi-Institutional Study J Am Coll Surg 2018 226 05 796 803 29454101 10.1016/j.jamcollsurg.2018.01.053

[12] Baumgartner C Maselli J Auerbach A D Fang M C Aspirin compared with anticoagulation to prevent venous thromboembolism after knee or hip arthroplasty: a large retrospective cohort study J Gen Intern Med 2019 34 10 2038 2046 31236894 10.1007/s11606-019-05122-3 PMC6816584

[13] Sun G Wu J Wang Q Factor Xa inhibitors and direct thrombin inhibitors versus low-molecular-weight heparin for thromboprophylaxis after total hip or total knee arthroplasty: A systematic review and meta-analysis J Arthroplasty 2019 34 04 789 8.0E8 30685261 10.1016/j.arth.2018.11.029

[14] Deirmengian G K Heller S Smith E B Maltenfort M Chen A F Parvizi J Aspirin can be used as prophylaxis for prevention of venous thromboembolism after revision hip and knee arthroplasty J Arthroplasty 2016 31 10 2237 2240 27118182 10.1016/j.arth.2016.03.031

[15] Anderson D R Dunbar M Murnaghan J Aspirin or rivaroxaban for VTE prophylaxis after hip or knee arthroplasty N Engl J Med 2018 378 08 699 707 29466159 10.1056/NEJMoa1712746

[16] Farey J E An V VG Sidhu V Karunaratne S Harris I A Aspirin versus enoxaparin for the initial prevention of venous thromboembolism following elective arthroplasty of the hip or knee: A systematic review and meta-analysis Orthop Traumatol Surg Res 2021 107 01 102606 32631716 10.1016/j.otsr.2020.04.002

[17] Venker B T Ganti B R Lin H Lee E D Nunley R M Gage B F Safety and efficacy of new anticoagulants for the prevention of venous thromboembolism after hip and knee arthroplasty: A meta-analysis J Arthroplasty 2017 32 02 645 652 27823844 10.1016/j.arth.2016.09.033 PMC5258767

[18] Le G Yang C Zhang M Efficacy and safety of aspirin and rivaroxaban for venous thromboembolism prophylaxis after total hip or knee arthroplasty: A protocol for meta-analysis Medicine (Baltimore) 2020 99 49 e23055 33285683 10.1097/MD.0000000000023055 PMC7717737

[19] Xu J Kanagaratnam A Cao J Y Chaggar G S Bruce W A comparison of aspirin against rivaroxaban for venous thromboembolism prophylaxis after hip or knee arthroplasty: A meta-analysis J Orthop Surg (Hong Kong) 2020 28 01 2.309499019896024E15 10.1177/2309499019896024 31908175

[20] French Society of Orthopaedic Surgery, Traumatology (SofCOT) Jenny J Y Bulaid Y Boisrenoult P Bleeding and thromboembolism risk of standard antithrombotic prophylaxis after hip or knee replacement within an enhanced recovery program Orthop Traumatol Surg Res 2020 106 08 1533 1538 33127330 10.1016/j.otsr.2020.02.026

[21] Wilson D G Poole W E Chauhan S K Rogers B A Systematic review of aspirin for thromboprophylaxis in modern elective total hip and knee arthroplasty Bone Joint J 2016 98-B 08 1056 1061 27482017 10.1302/0301-620X.98B8.36957

[22] Lassen M R Gent M Kakkar A K The effects of rivaroxaban on the complications of surgery after total hip or knee replacement: results from the RECORD programme J Bone Joint Surg Br 2012 94 11 1573 1578 23109641 10.1302/0301-620X.94B11.28955

[23] Kulshrestha V Kumar S DVT prophylaxis after TKA: routine anticoagulation vs risk screening approach - a randomized study J Arthroplasty 2013 28 10 1868 1873 23796558 10.1016/j.arth.2013.05.025

[24] Singh V Shahi A Saleh U Tarabichi S Oliashirazi A Persistent wound drainage among total joint arthroplasty patients receiving aspirin vs coumadin J Arthroplasty 2020 35 12 3743 3746 32788061 10.1016/j.arth.2020.07.004

[25] Garfinkel J H Gladnick B P Roland N Romness D W Increased incidence of bleeding and wound complications with Factor-Xa inhibitors after total joint arthroplasty J Arthroplasty 2018 33 02 533 536 28947374 10.1016/j.arth.2017.08.039

[26] Parvizi J Huang R Restrepo C Low-dose aspirin is effective chemoprophylaxis against clinically important venous thromboembolism following total joint arthroplasty: A preliminary analysis J Bone Joint Surg Am 2017 99 02 91 98 28099298 10.2106/JBJS.16.00147

[27] Peraturan Menteri Kesehatan Republik Indonesia. (n.d.)Available from:www.inaproc.lkpp.go.id; [accessed October 17, 2022].

