# Supplementary material for: The Comparison of Aspirin and Direct Oral Anticoagulant as Thromboprophylaxis Following Total Knee Replacement: A Retrospective Study
Source: Rev Bras Ortop (Sao Paulo). 2024 Dec 21;59(6):e883–7. doi: 10.1055/s-0044-1785510 (PMC11663063; doi:10.1055/s-0044-1785510)
Supplement: Related texts

# Research article: Comparação de ácido acetilsalicílico e anticoagulante oral direto como tromboprofilaxia após artroplastia total do joelho: Um estudo retrospectivo

**Keywords:** anticoagulantes, artroplastia do joelho, tromboembolia venosa

## Abstract

**Objetivo** O tromboembolismo venoso (TEV) ainda é um grande desafio após grandes cirurgias ortopédicas, incluindo a artroplastia total do joelho (ATJ). O objetivo deste estudo foi estimar o risco de TEV com profilaxia farmacológica apenas com ácido acetilsalicílico ou anticoagulante oral direto (AOD) após a cirurgia primária de ATJ.

**Métodos** O estudo incluiu 476 pacientes submetidos a ATJ primária de 2016 a 2020. Todos os pacientes receberam tromboprofilaxia com AOD (grupo AOD) (n = 267) ou ácido acetilsalicílico (AAS) (grupo AAS) (n = 209). Os desfechos clínicos foram avaliados e comparados entre aqueles que receberam AOD e AAS. O desfecho primário foi a incidência de TEV. O desfecho secundário foi a ocorrência de complicações da ferida.

**Resultado** O AAS e o AOD foram comparáveis na prevenção de TEV em pacientes submetidos a ATJ primária. A incidência de trombose venosa profunda foi semelhante nos grupos AAS (10%) e AOD (10,1%, *p* = 0,98), sem nenhum caso de embolia pulmonar em ambos os grupos. Não houve diferença significativa entre os grupos AAS (1,4%) e AOD (1,5%) em relação às complicações da ferida ( *p* = 0,95).

**Conclusão** A tromboprofilaxia pós-operatória apenas com AAS não foi associada a um maior risco de TEV pós-operatório em comparação à AOD após ATJ. Considerando a ampla disponibilidade e custo-benefício, o AAS pode ser uma alternativa promissora ao AOD para a profilaxia de TEV.

## Introdução

A atroplastia total do joelho (ATJ) é uma das intervenções ortopédicas de maior sucesso e impacto na melhoria da qualidade de vida. Estudos de projeção sugerem que a demanda por ATJ primária aumentará constantemente nos próximos anos. 1 No entanto, o procedimento de ATJ também apresenta algumas complicações pós-operatórias. Uma das complicações mais comuns, mas grave, é o tromboembolismo venoso (TEV). O TEV é uma complicação conhecida das cirurgias ortopédicas de grande porte, incluindo artroplastia de joelho ou quadril. 2 3

Diversos estudos examinaram a eficácia de vários medicamentos antitrombóticos ao longo dos anos, bem como seus perfis de efeitos colaterais, inclusive exsudação prolongada de feridas, sangramento e infecção. 4 5 Os agentes mais usados para tromboprofilaxia são antiplaquetários (ácido acetilsalicílico [AAS]) ou anticoagulantes orais diretos (AOD), como os inibidores do fator Xa (rivaroxabana ou edoxabana). Contudo, reconhece-se hoje que o AAS é preferível a outros anticoagulantes devido ao seu baixo risco de hemorragia grave. 6 7 O AAS pode ser um agente tromboprofilático alternativo após a ATJ primária ou artroplastia primária do quadril, em especial em indivíduos com risco elevado de hemorragia. Outras vantagens do AAS sobre os AODs incluem a facilidade de administração, a ausência de necessidade de monitoramento e o baixo custo. 8

A Indonésia é um dos países de renda média-baixa de acordo com o Banco Mundial. 9 Portanto, é necessário encontrar a tromboprofilaxia mais eficaz e segura com o custo mais razoável. O presente estudo tem como objetivo avaliar a eficácia clínica e segurança do AAS em comparação ao AOD para profilaxia de TEV após ATJ em uma única instituição. Nossa hipótese é que não há diferença significativa entre AAS e AOD na prevenção de TEV após procedimento de ATJ.

## Métodos

## Fonte de dados e coorte do estudo

O Comitê de Ética de nossa instituição aprovou este estudo sob o número 940/RSSC-5B/AL/IX/DIRUT/2022. Dados retrospectivos de 476 ATJs primárias foram extraídos de cinco anos (2016–2020) de relatos anuais de dados de registro de ATJ em nossa instituição. Os critérios de inclusão foram os seguintes: (1) pacientes submetidos à cirurgia primária de ATJ; e (2) pacientes que receberam AAS ou AOD para profilaxia de TEV. Os critérios de exclusão foram os seguintes: (1) doença hemorrágica prévia; (2) histórico de estado de hipercoagulação; (3) histórico de malignidade; (4) histórico de insuficiência venosa; (5) ATJ bilateral; (6) componente tibial posterior não metálico. A Fig. 1 mostra a seleção da amostra do estudo.

**Fig. 1:** Fluxograma de seleção da amostra do estudo. AAS, ácido acetilsalicílico; ATJ, artroplastia total de joelho; AOD, anticoagulante oral direto.

O desfecho primário foi TEV sintomático pós-operatório ocorrido durante a internação índice ou responsável por readmissão dentro de 30 dias ou consulta ambulatorial em 90 dias após a alta porque o aumento do risco tromboembólico persiste até 3 meses de pós-operatório. O desfecho secundário foi a ocorrência de complicação da ferida. Todos os pacientes foram submetidos à mesma técnica cirúrgica (abordagem parapatelar medial e aplicação de torniquete com 150 mmHg acima da pressão arterial diastólica) e protocolo perioperatório (medicação para dor perioperatória e 2 g de cefazolina intravenosa como antibiótico profilático). A profilaxia mecânica consistiu no bombeamento periódico do tornozelo com início 6 horas após a cirurgia e a aplicação de dispositivo de compressão pneumática intermitente (DICP). Todos os pacientes receberam tromboprofilaxia química com 160 mg de AAS (grupo AAS) ou inibidores do fator Xa (grupo AOD), ou seja, 10 mg de rivaroxabana (Xarelto; Bayer Pharmaceutical, Leverkusen, Alemanha) ou 30 mg de edoxabana (Lixiana; Kalbe Farma, Jacarta, Indonésia) 12 horas após a cirurgia e mantida por 14 dias. Todos os implantes utilizados neste estudo foram PFC Sigma ou Attune (DePuy Synthes, Raynham, MA, EUA). As investigações para detecção de trombose venosa profunda (TVP) foram iniciadas em pacientes sintomáticos no exame de acompanhamento de rotina. O diagnóstico de TEV foi baseado na confirmação da TVP na veia poplítea ou nas veias mais proximais da perna (incluindo as veias femoral, femoral comum e ilíaca) por ultrassonografia realizada por um radiologista. A complicação da ferida foi definida pela necessidade de irrigação e desbridamento, com ou sem troca de componentes. Todos os dados foram coletados em prontuários por dois médicos do serviço de ortopedia que não participaram da cirurgia.

## Análise estatística

Todos os dados experimentais foram analisados estatisticamente utilizando o software SPSS *Statistics for Windows* , versão 26.0 (IBM Corp, Armonk, NY, EUA). A análise bivariada foi utilizada para testar a associação entre a tromboprofilaxia individual e os principais desfechos clínicos de TEV pós-operatório por meio do teste qui-quadrado. A significância estatística foi estabelecida em *p* < 0,05.

## Resultado

A Tabela 1 mostrou a ausência de diferenças demográficas significativas entre os dois grupos. Os 2 grupos eram compostos majoritariamente por mulheres (80% *versus* 81%). Mais de ⅔ dos pacientes em ambos os grupos tinham comorbidades. Ambos os grupos apresentam perfis de comorbidades semelhantes, sendo a hipertensão a comorbidade mais comum (45,4% e 51,3% no grupo AAS e no grupo AOD, respectivamente). O tempo de internação nos grupos AAS e AOD foi equivalente (4,4 *versus* 4,6 dias).

**Tabela 1:** Dados demográficos dos pacientes

Característica	Grupo AAS N = 209 (45%)	Grupo AOD N = 267 (55%)	Valor de *p*
Idade média (anos)	68,3	67,9	0,32
Sexo: masculino/feminino (%)	43/166 (20/80)	53/214 (19/81)	0,06
Comorbidades			0,94
Hipertensão (%)	95 (45,4)	137 (51,3)	
Diabetes mellitus tipo 2 (%)	32 (15,3)	39 (14,6)	
Doença cardiovascular (%)	29 (13,8)	28 (10,4)	
Doença renal (%)	7 (3,3)	2 (0,7)	
Doença cerebrovascular (%)	3 (1,4)	3 (1,1)	
Doença hepática (%)	3 (1,4)	3 (1,1)	
Tempo médio de internação (desvio-padrão)	4,4 (2,7)	4,6 (2,1)	0,09

Abreviaturas: AAS, ácido acetilsalicílico; AOD, anticoagulante oral direto.

Houve 21 casos de TVP (10%) no grupo AAS em comparação a 27 casos (10,1%) no grupo AOD ( *p* = 0,98). Complicações da ferida foram observadas em 3 casos do grupo AAS (1,4%) e 4 casos do grupo AOD (1,5%) ( *p* = 0,95). Apenas dois casos necessitaram de troca de componentes devido a infecção articular protética, um de cada grupo. Os demais casos foram tratados com irrigação e desbridamento. A Fig. 2 mostra o número semelhante de incidência de TVP e complicações da ferida após a ATJ entre os grupos AAS e AOD.

**Fig. 2:** Incidência de trombose venosa profunda (TVP) e complicações da ferida nos grupos tratados com ácido acetilsalicílico (AAS) ou anticoagulante oral direto (AOD).

## Discussão

Nosso estudo revela que o AAS e o AOD (rivaroxabana ou edoxabana) foram comparáveis na prevenção de TEV em pacientes submetidos à ATJ. A incidência de TVP foi semelhante nos 2 grupos (10% versus 10,1%, respectivamente), sem ocorrência de êmbolos pulmonares (EP) em ambos os grupos. Não houve associação entre o tipo de agente profilático de TEV com o risco de complicações da ferida em pacientes pós-ATJ porque as incidências nos grupos AAS e AOD (1,4% *versus* 1,5%, respectivamente) também foram semelhantes.

A incidência exata de TVP e EP ainda é controversa. 10 Sem qualquer protocolo de profilaxia, as taxas de incidência foram superiores a 50% após a artroplastia. 11 A incidência diminuiu drasticamente graças à mobilização precoce, uso de dispositivos de compressão mecânica e profilaxia química. 11 Há um debate substancial sobre o agente profilático químico preferido (AAS, enoxaparina, varfarina e AOD) ao avaliar a eficácia da profilaxia de TEV em relação ao risco de sangramento. 12 Os AODs são melhores na profilaxia de TEV em comparação a outros anticoagulantes, com menor taxa de complicações, como a necessidade de transfusão de sangue e drenagem prolongada de feridas. 13 O AAS é bastante utilizado na prevenção primária ou secundária de doenças cardiovasculares pois inibe a agregação plaquetária pela acetilação irreversível da cicloxigenase, interferindo assim na capacidade de produção de prostaglandinas pelas plaquetas. O AAS também foi comparável ao AOD na profilaxia de TEV. 12 14 Recentemente, alguns estudos recomendaram o uso de AAS como opção para profilaxia de TEV, além de AOD ou heparina de baixo peso molecular (HBPM) na artroplastia eletiva, incluindo ATJ, combinado à profilaxia mecânica até a alta hospitalar. 15

Este estudo mostrou que a incidência de TEV não foi influenciada pelo tipo de tromboprofilaxia utilizada, o que é corroborado pela literatura recente. Diversos estudos também concluíram que o uso de AAS após a ATJ estava associado a baixas taxas de eventos tromboembólicos, semelhantes às taxas observadas nos pacientes tratados com AOD. 14 15 O AAS leva a uma baixa taxa de TEV, sem diferença em comparação à enoxaparina após a ATJ. 16 Venker et al. 17 mostraram que o AOD foi superior à enoxaparina na prevenção da TVP. Outros estudos clínicos randomizados de Le et al. 18 e Xu et al. 19 relataram que não houve diferença entre AOD (rivaroxabana) e AAS na profilaxia de TEV após a ATJ, consistente com nossos achados. A administração de profilaxia para TEV também apresenta várias desvantagens, como sangramento pós-operatório e drenagem prolongada da ferida. 19 O sangramento pós-operatório é uma das principais preocupações da profilaxia da TEV. Quanto mais potente o agente farmacológico, maior o risco que representa. 20 Descobriu-se que o AAS apresenta menor risco de sangramento comparado com outros agentes para profilaxia de TEV, como relatado por Wilson et al. 21 Da mesma forma, o AOD também apresenta baixo risco de sangramento em comparação à enoxaparina e varfarina, especialmente se administrado em dose baixa. 13 Outra desvantagem da profilaxia de TEV são as complicações pós-operatórias da ferida. Um estudo conduzido por Lassen et al. 22 relatou que a administração de rivaroxabana aumentou as complicações da ferida após a ATJ, embora essas complicações não tenham sido tão graves quanto aquelas observadas em pacientes em uso de heparina de baixo peso molecular (HBPM), conforme relatado por Kulsrestha et al. 23 Singh et al. 24 demonstraram que o uso de AAS reduz a taxa de drenagem prolongada da ferida em pacientes pós-ATJ, diminuindo, portanto, o risco de desenvolvimento de qualquer possível complicação da ferida, incluindo infecção superficial e profunda. Da mesma forma, outro estudo de Garfinkel et al. 25 relatou que pacientes que receberam AOD, como apixabana, para profilaxia de TEV após a artroplastia, apresentaram taxa significativamente maior de complicações da ferida e sangramento. Curiosamente, nosso estudo descobriu que não houve diferença estatística entre AAS e AOD em termos de complicações da ferida.

O AAS é um medicamento antiplaquetário genérico, de custo baixo e ampla disponibilidade. Portanto, o uso de AAS como profilaxia de TEV pode ser uma escolha com boa relação custo-benefício. 26 Como já afirmado, a Indonésia ainda está entre os países de renda média-baixa, de acordo com a Organização Mundial da Saúde. O catálogo eletrônico do Ministério da Saúde da Indonésia cita que o custo do AAS é de Rp 116,-/comprimido (0,79 ¢), enquanto o preço da rivaroxabana é de Rp 23.500,-/comprimido ($ 1,39). 2 7 Logo, dado o efeito comparável do AOD como agente de tromboprofilaxia, o AAS continua a ser o agente trombofilático de melhor custo-benefício, especialmente quando comparado ao inibidor do fator Xa.

Este estudo apresenta algumas limitações. Primeiro, por ser um estudo retrospectivo, a força da evidência é menor. Os resultados ainda precisam ser confirmados com futuros ensaios prospectivos e maior nível de evidência. Em segundo lugar, existem seleções inerentes e vieses do observador, uma vez que os pacientes eram de um único hospital. Apesar dessas limitações, nossos achados são clinicamente importantes. As amostras em nosso estudo são grandes e, assim, podem ser adequadas para mostrar que o AAS não é inferior em comparação à rivaroxabana. Futuros ensaios multicêntricos são necessários para reduzir os vieses.

## Conclusão

Pacientes submetidos a cirurgias ortopédicas de grande porte, como ATJ, apresentam maior risco de desenvolver TEV durante e após a hospitalização. O presente estudo mostrou que não houve diferença significativa entre AAS e AOD na prevenção de TEV após ATJ. Na escolha da profilaxia para TEV em pacientes submetidos à cirurgia de ATJ, dada a segurança, ampla disponibilidade e custo-efetividade, o AAS pode ser considerado uma alternativa promissora ao AOD para a profilaxia de TEV.
